# Functional characterization of the AGL1 aegerolysin in the mycoparasitic fungus *Trichoderma atroviride* reveals a role in conidiation and antagonism

**DOI:** 10.1007/s00438-020-01732-3

**Published:** 2020-10-14

**Authors:** Mukesh Dubey, Dan Funck Jensen, Magnus Karlsson

**Affiliations:** Department of Forest Mycology and Plant Pathology, Uppsala Biocenter, Swedish University of Agricultural Sciences, P.O. Box 7026, 75007 Uppsala, Sweden

**Keywords:** Aegerolysin, Antagonism, Haemolysin, Mycoparasitism, Pore-forming proteins, Trichoderma

## Abstract

Aegerolysins are small secreted pore-forming proteins that are found in both prokaryotes and eukaryotes. The role of aegerolysins in sporulation, fruit body formation, and in lysis of cellular membrane is suggested in fungi. The aim of the present study was to characterize the biological function of the aegerolysin gene *agl1* in the mycoparasitic fungus *Trichoderma atroviride*, used for biological control of plant diseases. Gene expression analysis showed higher expression of *agl1* during conidiation and during growth in medium supplemented with cell wall material from the plant pathogenic fungus *Rhizoctonia solani* as the sole carbon source. Expression of *agl1* was supressed under iron-limiting condition, while *agl1* transcript was not detected during *T*. *atroviride* interactions with the prey fungi *Botrytis cinerea* or *R*. *solani*. Phenotypic analysis of *agl1* deletion strains (Δ*agl1*) showed reduced conidiation compared to *T*. *atroviride* wild type, thus suggesting the involvement of AGL1 in conidiation. Furthermore, the Δ*agl1* strains display reduced antagonism towards *B. cinerea* and *R*. *solani* based on a secretion assay, although no difference was detected during direct interactions. These data demonstrate the role of AGL1 in conidiation and antagonism in the mycoparasitic fungus *T*. *atroviride*.

**Electronic supplementary material:**

The online version of this article (10.1007/s00438-020-01732-3) contains supplementary material, which is available to authorized users.

## Introduction

The aim of this study was to characterize the biological function of an aegerolysin protein in the fungus *Trichoderma atroviride*, used for biological control of plant pathogenic fungi in agriculture, with emphasis on its role in asexual development and interactions with other fungi. Aegerolysin (Pfam 06355, InterPro IPR009413) is a family of lipid-binding proteins that are found in both prokaryotes and eukaryotes (Novak et al. 2015; Butala et al. 2017). Aegerolysin family proteins are typically small (15–20 kDa) with low isoelectric point, a predicted β-structure and are shown to be active in a broad pH range from 3.5 to 10.5 (Novak et al. 2015; Butala et al. 2017). In fungi, aegerolysins are reported from many species with varying lifestyles and ecological niches; however, functional characterization is only reported from a few species. In the basidiomycete fungi *Agrocybe aegerita*, *A. cylindracea*, *Pleurotus ostreatus*, and *P. eryngii*, expressions of aegerolysin genes are up-regulated during sexual development, specifically during the development of young fruiting bodies (primordia) and in the basidiospores (Fernandez Espinar and Labarère 1997; Berne et al. 2002; Lee et al. 2002; Shim et al. 2006; Joh et al. 2007). Likewise, the expressions of aegerolysin genes are induced during asexual development, in the conidiophore and conidia of the ascomycetes *Alternaria gaisen* and *Aspergillus oryzae*, respectively (Bando et al. 2011; Roberts et al. 2011). Higher expressions of aegerolysin genes are also recorded during spore germination and hyphal growth of *A. fumigatus*, *A. terreus*, and *A. niger* (Wartenberg et al. 2011; Nayak et al. 2012, 2019). However, deletions of the aegerolysin genes *nigA1* and *nigA2* in the plant pathogen *A*. *niger* do not affect growth, sporulation, or virulence in the corresponding mutants, suggesting functional redundancy (Novak et al. 2019). In addition, expressions of *nigA1* and *nigA2* are shown to be up-regulated by nutrient source (carbon and nitrogen), abiotic stress (temperature and pH), oxidative stress (H_2_O_2_), and anaerobic stress (Novak et al. 2019).

Aegerolysin family proteins are haemolytic and cytolytic to mammalian cell lines and insect cells (Butala et al. 2017; Panevska et al. 2019). To exert cytolysis, certain aegerolysins interact with the cellular membrane in their monomeric form; for example, AspHS from *A*. *fumigatus*. While others, for example ostreolysin A (OlyA), pleurotolysin A2 (PlyA2), and erylysin A (EryA) from *P*. *ostreatus*, function in their heterodimeric form in combination with other proteins carrying the membrane attack complex/perforin domain, such as pleurotolysin B (PlyB) and erylysin B (EryB) (Tomita et al. 2004; Shibata et al. 2010; Gilbert et al. 2013; Ota et al. 2013; Panevska et al. 2019).

The aegerolysin protein asp-haemolysin (AspHS) from the human pathogen *A*. *fumigatus* is shown to lyse mammalian cell lines, consequently decreasing the viability of macrophages and human vascular endothelial cells (Kumagai et al. 1999, 2001) and thereby contributing to *A*. *fumigatus* virulence. However, functional characterization of AspHS by genetic transformation results in no significant difference in virulence between the wild type (WT) and the *aspHS* deletion mutant, suggesting functional redundancy of the AspHS aegerolysin in *A*. *fumigatus*. Furthermore, purified aegerolysins (OlyA, PlyA2, and EryA) from *P*. *ostreatus* exhibited toxicity towards western corn rootworm larvae and adults and Colorado potato beetle larvae by enhancing the permeability of the insect cell membrane (Panevska et al. 2019). The toxic effect of aegerolysins is a result of their interactions with cell membrane lipids that induce the formation of trans-membrane pores and lead to the disruption of membrane permeability and osmotic lysis (Nayak et al. 2011, 2015; Butala et al. 2017; Panevska et al. 2019).

*Trichoderma atroviride* is a filamentous ascomycete fungus that can parasitize and kill several plant pathogenic fungi (mycoparasitism), and certain strains are therefore used commercially as biological control agents against plant pathogenic fungi in agricultural and horticultural production systems (Druzhinina et al. 2011). *T*. *atroviride* produces hydrolytic enzymes and secondary metabolites as part of the mycoparasitic attack on the fungal prey (Druzhinina et al. 2011; Mukherjee et al. 2012).

In the current study, we identified a single aegerolysin gene (*agl1*) in the *T. atroviride* genome. Our results showed an induced expression of *agl1* in conidiating mycelia, and during hyphal growth on medium supplemented with cell wall material from the plant pathogenic fungus *Rhizoctonia solani*. By generating *agl1* deletion mutants, we demonstrated a role for AGL1 in conidial development and antagonism in *T*. *atroviride*.

## Materials and methods

### Gene identification, sequence analysis, and phylogenetic analysis

Genome sequences of 100 fungal species were screened for the presence of aegerolysin genes by BLASTP analysis using amino acid sequences of characterized aegerolysins from the ascomycetes *A. fumigatus* (GenBank accession number: EAL86341) and *A. terreus* (EAU36830), and the basidiomycetes *A. aegerita* (AAC02265), *P. eryngii* (BAN83907), and *P. ostreatus* (AAX21097). The presence of signal peptides and conserved domains were analysed using the Simple Modular Architecture Research Tool (SMART) (Bork et al. 2009) and Conserved Domain Search (CDS) (Marchler-Bauer et al. 2011). SecretomeP 2.0 (Bendtsen et al. 2004) was used to search for non-classical secretion signals.

Alignment of aegerolysin amino acid sequences was done using Muscle (Edgar 2004) and phylogenetic analysis was performed using maximum-likelihood methods implemented in MEGA X ver. 10 (Kumar et al. 2018). The WAG (Whelan and Goldman) with G (Gamma distribution) amino acid substitution model (Whelan and Goldman 2001) was used and pairwise deletion of gaps. Statistical support for branches was assessed by 1000-iteration bootstrap resampling.

### Fungal strains and culture conditions

*T*. *atroviride* strain IMI206040 (WT) and mutants derived from it, *Botrytis cinerea* strain B05.10 and *R. solani* strain SA1 were maintained on potato dextrose agar (PDA; Oxoid, Cambridge, UK) medium at 25 °C. Synthetic minimal salt (SMS) liquid medium (Dubey et al. 2012) supplemented with 1% glucose was used for submerged liquid culture unless otherwise specified. Culture medium for different nutrient conditions was prepared by substituting 1% glucose in SMS medium with colloidal chitin (1%), *R*. *solani* cell wall material (RsCW) (1%), or *N*-acetylglucosamine (NAG; Sigma-Aldrich, St. Louis, MO, USA) (10 mM). Limitation media for carbon (C lim), nitrogen (N lim), and carbon + nitrogen (C + N lim) were prepared as described before (Dubey et al. 2012). Starvation for iron (Fe lim) was induced by a tenfold reduction of FeSO_4_ × 7H_2_O concentration in SMS medium. Colloidal chitin was prepared from crab-shell chitin (Sigma-Aldrich, St. Louis, MO, USA) as described previously (Roberts and Selitrennikoff 1988). *R. solani* cell wall material was prepared as described previously (Inglis and Kawchuk 2002).

### Gene expression analysis

Gene expression analysis of *agl1* in *T*. *atroviride* was performed during growth in liquid media supplemented with different nutrients or stress agents, during mycoparasitic fungal–fungal interactions with *B. cinerea* and *R*. *solani*, and during different developmental stages.

For gene expression analysis in different nutritional conditions, *T*. *atroviride* mycelium was cultivated in 50 ml SMS medium with 1% glucose in flasks (Dubey et al. 2012), and harvested by filtering through Miracloth, washed with sterile distilled water, and transferred to new flasks containing 50 ml of fresh SMS medium containing different nutrient regimes. *T*. *atroviride* cultivated in SMS with 1% glucose was used as control treatment. Mycelia were harvested 24 h post-inoculation (hpi), washed in distilled sterile water, frozen in liquid nitrogen, and stored at − 70 °C.

For gene expression analysis during interactions, an in vitro dual culture plate confrontation assay was used (Dubey et al. 2012). A 3 mm-diameter agar plug cut from the growing front of *T*. *atroviride* and *B*. *cinerea* or *R*. *solani* mycelia was inoculated on opposite sides of a 9 cm-diameter PDA plate, covered with a cellophane membrane to facilitate harvesting of the mycelium. The mycelial front (7–10 mm) of *T*. *atroviride* from the interaction zone was harvested 24 h after contact and immediately frozen in liquid nitrogen and stored at − 70 °C. *T*. *atroviride* confronted with itself was used as control treatment.

For gene expression analysis during different developmental stages, a 3 mm-diameter agar plug from the growing mycelial front of *T*. *atroviride* was transferred to PDA plates covered with a cellophane membrane. Experimental plates were incubated in darkness, except for a daily 30 min light treatment to induce conidiation. Mycelia were harvested 2 days post-inoculation (dpi) representing vegetative mycelial growth stage (VM), at 3 dpi representing the early stage of conidiating mycelium (ECM) and at 5 dpi representing conidiated mycelium (CM).

RNA extraction was done using the Qiagen RNeasy kit following the manufacturer’s protocol (Qiagen, Hilden, Germany). One microgram of total RNA was reverse transcribed (RT) in a total volume of 20 μl using the Maxima first strand cDNA synthesis kit (Fermentas, St. Leon-Rot, Germany). Transcript levels were estimated by quantitative PCR (qPCR) using the SYBR Green PCR Master Mix (Fermentas, St. Leon-Rot, Germany) in an iQ5 qPCR System (Bio-Rad, Hercules, CA, USA) as described previously (Dubey et al. 2012). Melt curve analysis was performed after the qPCR reactions, to confirm that the signal was the result of a single product amplification. Relative expression levels for *T*. *atroviride* target genes in relation to *act1* and *sar1* (Brunner et al. 2008) expression were calculated from the Ct values and the primer amplification efficiencies by using the formula described by Pfaffl (2001). Gene expression analysis was carried out in four biological replicates, each based on two technical replicates. Primer sequences used for gene expression analysis are given in Table 1.

**Table 1:** List of primers used in this study

Name	Target/purpose	Sequences(5′ → 3′)
AL F	*agl1*	tcaaaaacgccgccctatca
AL R		cctgacgccgcacctgag
Actin F	*act1*	ctcacatccttcgccaatcactc
Actin R		agcccagctgccatacacaag
Sar1 F	*sar1*	ggtgatgcgacagggctacg
Sar1 R		tgtcatcaccgggagccacta
AL_ups F	*agl1* upstream	ggggacaactttgtatagaaaagttggccgcattgcctagacttgtt^a^
AL_ups R		ggggactgcttttttgtacaaacttgatggcaagggtaatgggtat^a^
AL_ds F	*agl1* downstream	ggggacagctttcttgtacaaagtggttgggagtgcgagaaaagagg^a^
AL_ds R		ggggacaactttgtataataaagttggcattgcagagcgattggtt^a^
Hyg F	hygB cassette	gcgcgcaattaaccctcac
Hyg R		gaattgcgcgtacagaactcc
AL_ko F	Mutant validation	ctggaggcgccagagatta
AL_ko R		gacgtagcccgggttgaaa

^a^attB and attBr sequences are underlined

### Construction of gene deletion cassette

Genomic DNA from *T. atroviride* was extracted using hexadecyl-tri-methyl-ammonium bromide (CTAB) method (Nygren et al. 2008). Phusion DNA polymerase (Finnzymes, Vantaa, Finland) was used for PCR amplification (Dubey et al. 2012) of ~ 1 kb 5′-flank and 3′-flank regions of the *agl1* gene from genomic DNA of *T*. *atroviride* using primer pairs AL_ups F/AL_ups R and AL_ds F AL_ds R, respectively (Table 1). Gateway entry clones of the purified 5′-flank and 3′-flank PCR fragments were generated as described by the manufacturer (Invitrogen, Carlsbad, CA, USA). The gateway entry clone for the hygromycin cassette (hygB), constructed during our previous studies (Dubey et al. 2020), was used. The gateway LR recombination reaction was performed using entry plasmid of respective fragments and the destination vector pPm43GW (Karimi et al. 2005) to generate the deletion vectors following the conditions described by the manufacturer (Invitrogen, Carlsbad, CA, USA).

### *Agrobacterium tumefaciens*-mediated transformation and validation of transformants

The deletion vector was transformed into *Agrobacterium tumefaciens* strain AGL1 based on a previous protocol for *T*. *harzianum* (Utermark and Karlovsky 2008). Transformed strains were selected on plates containing 100 µg/ml of hygromycin (Sigma-Aldrich, St. Louis, MO, USA). Validation of homologous integration of the deletion cassettes in putative transformants was performed using a PCR screening approach with primer combinations targeting the hygB cassette (Hyg F/Hyg R) and sequences flanking the deletion cassettes (AL_ko F/AL_ko R) as described previously (Dubey et al. 2020). PCR-positive transformants were repeatedly sub-cultured on PDA plates without the selectable agent five times, followed by re-exposure to selection antibiotics to test for mitotic stability. Two rounds of single spore purification were performed to purify mitotically stable colonies as described previously (Dubey et al. 2012). Expression of *agl1* in the WT and the gene deletion strains was determined by RT-PCR analysis using RevertAid premium reverse transcriptase (Fermentas, St. Leon-Rot, Germany) and primer pairs specific for *agl1* (AL F/AL R) (Table 1). Three independent *T*. *atroviride agl1* deletion strains (ΔA, Δ*agl1*B, and Δ*agl1*C) along with the WT were used in phenotype analyses.*agl1*

### Phenotypic analyses

A 3 mm-diameter agar plug from the growing mycelial front of *T. atroviride* strains was transferred to solid agar and colony morphology and growth diameter were recorded every day. Conidia were harvested from a 10 day old plate in 10 ml distilled water and filtered through Miracloth to remove the mycelial debris. Conidial concentration was determined under the microscope using a bright-line haemocytometer (Sigma-Aldrich, St. Louis, MO, USA). Each experiment had three biological replicates and was repeated two times.

### Antagonism tests

Antagonistic behaviour of *T*. *atroviride* WT and mutants against *B*. *cinerea* and *R*. *solani* was tested using an in vitro plate confrontation assay on PDA medium as described previously (Dubey et al. 2012). The growth of *B*. *cinerea* and *R*. *solani* was measured daily until their mycelial front reached the *T*. *atroviride* mycelial front, while the growth of *T*. *atroviride* strains was measured until the fungus reached the opposite side in the plate.

The role of secreted factors in antagonism was assayed by growing *T*. *atroviride* WT and mutant strains on PDA, covered with cellophane, at 25 °C. *T*. *atroviride* colonies were injured with a scalpel and exposed to light to induce conidiation (Hernández-Oñate et al. 2012). The cellophane was removed when *T*. *atroviride* covered the plates, followed by inoculation with *B*. *cinerea* or *R*. *solani* agar plugs. PDA plates that were not previously inoculated with *T*. *atroviride* strains were used as control. Linear growth of *B*. *cinerea* or *R*. *solani* was recorded 3 dpi. The experiment was performed in three biological replicates.

### Statistical analysis

Analysis of variance (ANOVA) was performed on gene expression and phenotype data using a General Linear Model approach implemented in Statistica version 13 (StatSoft, Tulsa, OK, USA). Pairwise comparisons were made using the Fisher’s or Tukey–Kramer methods at the 95% significance level.

## Results

### Identification and distribution of aegerolysin genes in Sordariomycota

Out of 100 analysed fungal genomes, representing 35 sordariomycete species, aegerolysin genes were identified in 26 genomes, representing 15 different species (Table S1). Analysis of 19 *Trichoderma* strains revealed that *T*. *asperellum* CBS 433.97, *T*. *virens* Gv29-8, and *T*. *atroviride* IMI 206040 contained one aegerolysin gene each (Table S1). Genome analysis of 12 strains representing six species with mycoparasitic behaviour from the genus *Clonostachys* showed the presence of one aegerolysin gene in all analysed genomes except for *Clonostachys solani* 1703 and *C. chloroleuca* CBS 570.77 that contained two genes each (Table S1). Among plant pathogenic *Fusaria*, a single aegerolysin gene was found in a single strain, *F*. *oxysporum* f. sp. *conglutinans* race 2 54008 (PHW808), among 15 different strains representing five species. No aegerolysin genes were identified in 11 different species of plant pathogenic *Colletotrichum* (Table S1). All investigated entomopathogenic species (*Beauveria*, *Cordyceps*, *Metarhizium,* and *Tolypocladium*) contained a single aegerolysin gene (Table S1).

### Sequence analysis of a predicted aegerolysin in *Trichoderma atroviride*

The identified aegerolysin gene in *T. atroviride* (protein ID: 32864) was 503 bp long with a 62 bp intron and named *agl1* (GenBank accession number: EHK42428.1). It was predicted to encode a polypeptide composed of 146 amino acids with a molecular weight of 15.98 kDa and an estimated isoelectric point at pH 6.72. SMART analysis predicted that AGL1 contained an aegerolysin domain (Pfam PF06355, InterPro IPR009413) between amino acid positions 8–140. This structure was further verified by CDS. No N-terminal secretion signal peptide was identified using SignalP. However, SecretomeP analysis predicted the secretion of AGL1 (score 0. 87) through a non-classical secretion pathway. Sequence alignments of the predicted aegerolysin proteins from the three *Trichoderma* species showed 66% and 48% amino acid identity between *T*. *atroviride* AGL1 and its homologues in *T*. *asperellum* and *T*. *virens*, respectively, while a 47% amino acid identity was found between *T*. *asperellum* and *T*. *virens*.

### Phylogenetic analysis

**Fig. 1:**
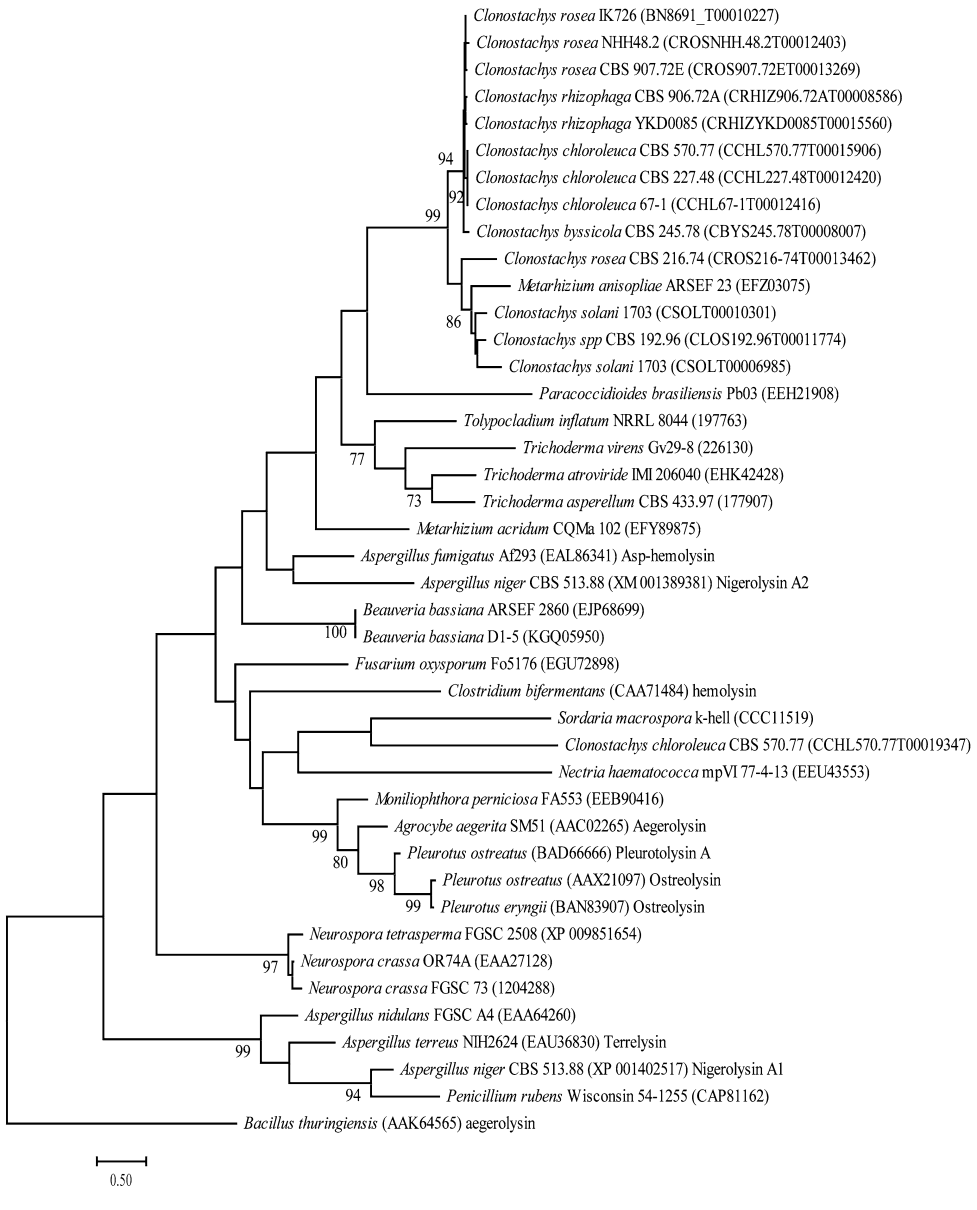
Phylogenetic analysis of fungal aegerolysins. Predicted amino acid sequences of fungal aegerolysins were aligned by muscle and used to construct a phylogenetic tree using maximum-likelihood methods in MEGA X ver. 10. Branch support values (bootstrap proportions ≥ 70%) are associated with nodes. Branch lengths indicate the average number of substitutions per site. Sequence identifiers include species name, GenBank ID number, or protein ID number from the respective genome project, and protein name (if available)

A phylogenetic analysis was conducted with *T*. *atroviride* AGL1, together with aegerolysins identified from other sordariomycetes (Table S1) and previously characterized aegerolysins. The result showed low resolution for the lower branches in the phylogenetic tree, but with several terminal groups of taxa with high (≥ 70%) bootstrap support (Fig. 1). Three examples of incongruence between the aegerolysin gene tree and the species relationships were identified; one *Metarhizium anisopliae* aegerolysin that clustered within a *Clonostachys* group, one *C. chloroleuca* aegerolysin that did not cluster with other *Clonostachys* aegerolysins, and one *Penicillium rubens* aegerolysin that clustered with *Aspergilli* aegerolysins (Fig. 1). All included basidiomycete aegerolysins formed a single monophyletic clade with 99% bootstrap support. AGL1 clustered together with the other two *Trichoderma* aegerolysins with 59% bootstrap support (Fig. 1).

### Gene expression analysis

**Fig. 2:**
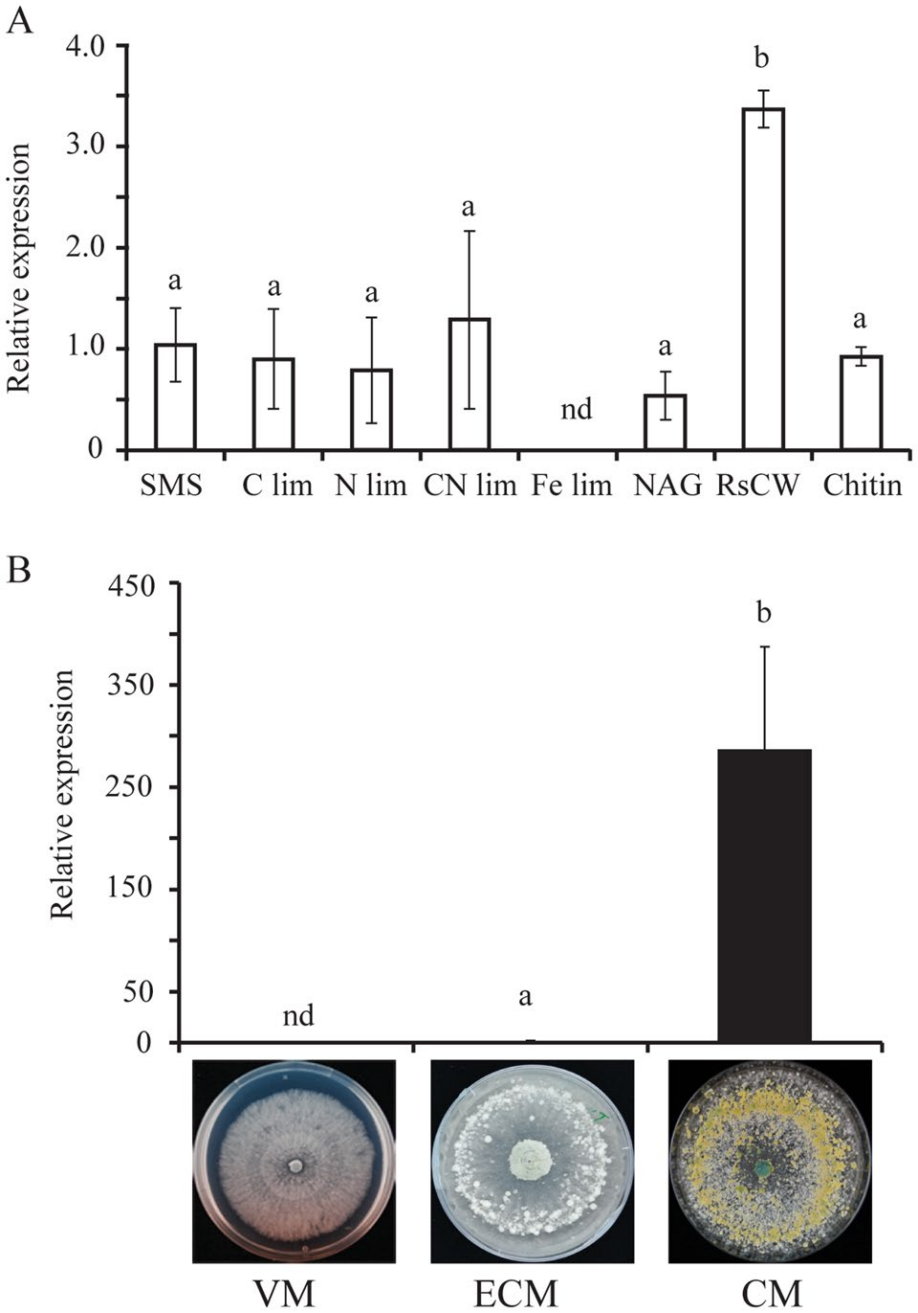
Expression analyses of *agl1* in *T*. *atroviride*. **a** Gene expression in mycelium grown in different nutritional/stress condition. **b** Gene expression analysis during different developmental stages. *VM* vegetative mycelia, *ECM* early stage of conidiation, *CM* conidiated mycelia. Relative expression levels for *agl1* in relation to *act1* were calculated from the Ct values and the primer amplification efficiencies using the formula described by Pfaffl (2001). Error bars represent standard deviation based on four biological replicates. Different letters indicate statistically significant differences (*P* ≤ 0.05) within experiments based on the Tukey–Kramer test

Gene expression of *agl1* was induced 3.2-fold (*P* < 0.001) in SMS medium supplemented with *R. solani* cell walls (RsCW) compared with the glucose control. In contrast, *agl1* expression was downregulated to a non-detectable level when *T*. *atroviride* was grown under iron-limited (Fe lim) condition (Fig. 2a). The *agl1* gene was also expressed in all other analysed culture media conditions, including liquid SMS supplemented with NAG or chitin, and conditions limited in carbon, nitrogen, and carbon + nitrogen (C lim, N lim, and C + N lim), although at the same level as the glucose control treatment. The expression of *agl1* was not detected during fungal–fungal interactions. During asexual development, transcript abundance of *agl1* was induced 281-fold (*P* < 0.001) in conidiating mycelium (CM) compared with the early stage of conidiation (ECM) (Fig. 2b). The expression of *agl1* was not detected during vegetative growth (VM).

### Generation and validation of *agl1* deletion mutants

*T*. *atroviride agl1* deletion mutants were generated by replacing *agl1* with the hygB cassette by *A. tumefaciens*-mediated transformation. Successful gene replacement in mitotically stable mutants was confirmed by PCR using primers located within the hygB cassette together with primers located upstream and downstream of the construct (Figure S1A) as described in our previous work (Dubey et al. 2020). The expected sizes of PCR fragments were amplified in all selected Δ*agl1* strains, while no amplification was observed in the WT (Figure S1B). Furthermore, RT-PCR experiments using primers specific to the *agl1* sequence demonstrated the complete loss of *agl1* transcript in each mutant (Figure S1C).

### Deletion of *agl1* did not affect growth rate and colony morphology

No difference in mycelial growth rate or colony morphology was found between the WT and Δ*agl1* deletion strains on PDA plates (Table S2). Although expression of *agl1* was up-regulated in SMS medium supplemented with RsCW, no difference in growth rate between *T*. *atroviride* WT and Δ*agl1* strains was detected on this medium (Table S2).

### Deletion of *agl1* results in decreased conidiation

**Fig. 3:**
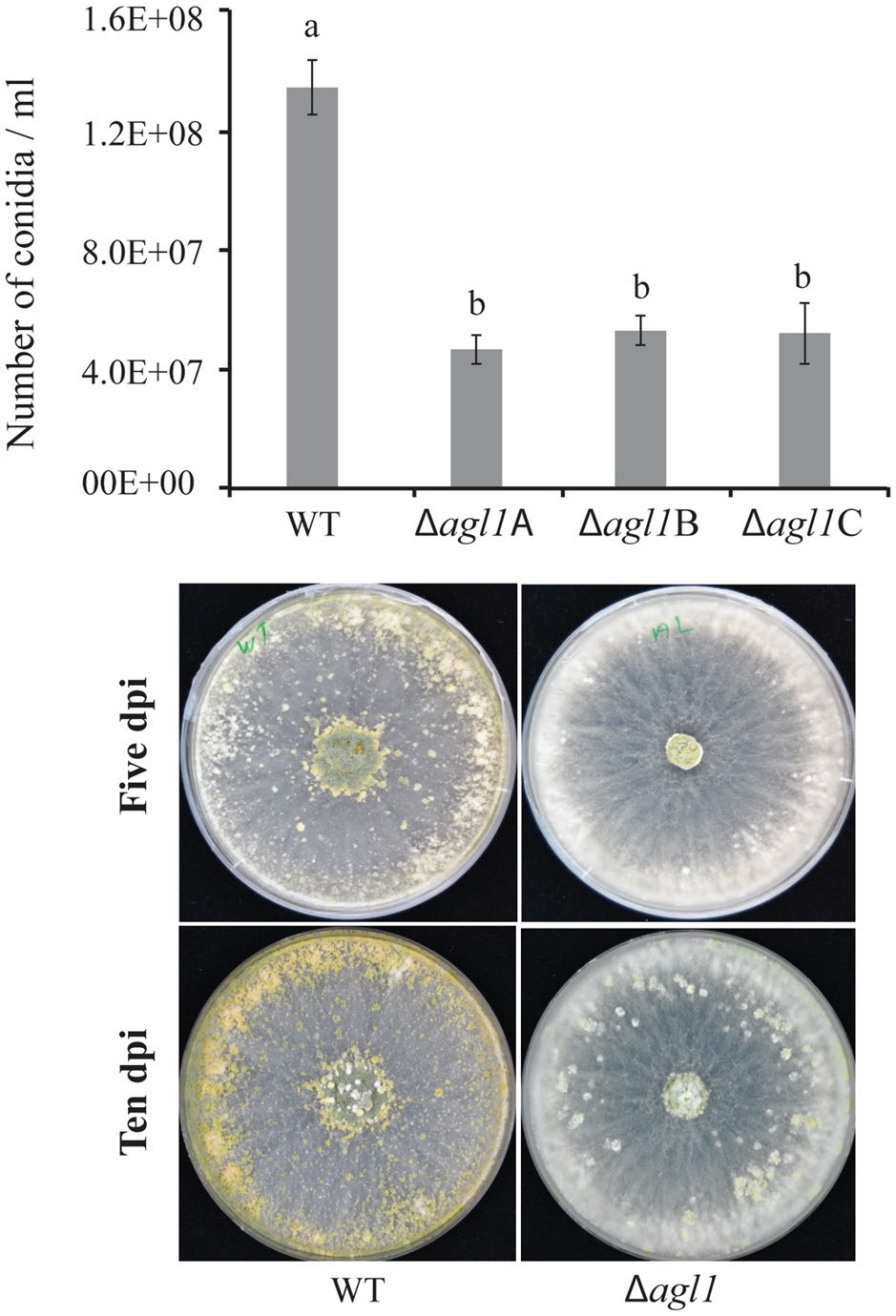
Conidiation in *T*. *atroviride* WT and Δ*agl1* strains on PDA medium. Conidia from the WT and deletion strains were harvested in equal volume of water and number was determined using a bright-line haemocytometer as per instruction of manufacturer. Error bars represent standard deviation based on three biological replicates. Different letters indicate statistically significant differences (*P* ≤ 0.05) within experiments based on the Tukey–Kramer test

Conidial production of *T*. *atroviride* WT and Δ*agl*1 strains was determined 10 dpi on PDA by counting the conidia harvested in equal amounts of water. The deletion strains showed on average a 2.9-fold lower (*P* < 0.001) conidiation in comparison to the WT (Fig. 3).

### Deletion of *agl1* reduces in vitro antagonism

**Fig. 4:**
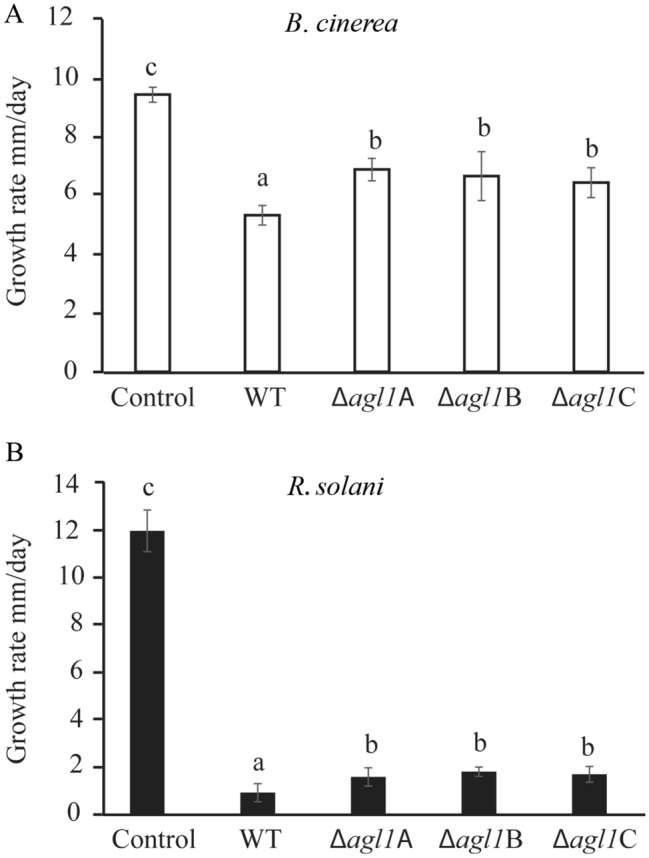
Secretion assay of *T. atroviride* strains. Agar plugs were inoculated on PDA plates covered with cellophane and incubated at 25 °C. After 3 dpi, the colony was removed together with the cellophane disc. Plates were re-inoculated with an **a** *B*. *cinerea* or **b** *R*. *solani* agar plug, incubated at 25 °C in darkness, and the growth diameter recorded at 3 dpi. *B*. *cinerea* or *R*. *solani* agar plugs inoculated on PDA plates that were not previously inoculated with *T*. *atroviride* strains were used as control. Error bars represent standard deviation based on three biological replicates. Different letters indicate statistically significant differences (*P* ≤ 0.05) within experiments based on Fisher’s exact test

During in vitro dual culture interaction, no differences in growth rate of *B*. *cinerea* or *R*. *solani* were recorded during confrontation with *T*. *atroviride* WT compared with Δ*agl1* strains (Table S3). Furthermore, no difference in the ability to overgrow *B*. *cinerea* and *R*. *solani* was observed between the WT and the deletion strains (data not shown). Since expression of *agl1* was highly induced in conidiating mycelia, *B. cinerea* and *R*. *solani* were grown on agar plates previously colonized by conidiating mycelia of *T*. *atroviride* WT or Δ*agl1* strains. Secretion assay showed significant reduction (*P* = 0.001) in growth rate of *B. cinerea* and *R*. *solani* on agar plates previously colonized by *T*. *atroviride* WT compared with control PDA plates (Fig. 4). Growth rate of *B. cinerea* and *R*. *solani* was 25% higher (*P* ≤ 0.038) and 87% higher (*P* ≤ 0.022), respectively, when grown on PDA plates previously colonized by Δ*agl1* strains compared with *T*. *atroviride* WT (Fig. 4). However, microscopic analysis showed no difference in hyphal/mycelial morphology of *B*. *cinerea* and *R*. *solani* grown on agar plates previously colonized by conidiating mycelia of *T*. *atroviride* WT or Δ*agl1* strains. Microscopic analysis indicates that the low growth rate of *B*. *cinerea* and *R*. *solani* on agar plates previously colonized by Δ*agl1* strains is not connected with deformation or lysis of hyphae.

## Discussion

The predicted structure of AGL1 in *T. atroviride*, with a single aegerolysin domain and the lack of a N-terminal secretion signal peptide, is similar to known aegerolysin proteins (Butala et al. 2017; Novak et al. 2019). We only identified aegerolysin genes in 26 out of 100 investigated sordariomycete genomes, which is in line with a previous study where putative aegerolysins was found to be present only in 22% of the fungal genomes sequenced until year 2014 (Novak et al. 2015). Taxonomic distribution of aegerolysin genes is further reported to be patchy across the fungal kingdom with low correlation to ecological niches or lifestyles (Novak et al. 2015; Butala et al. 2017), although we only identified two genera, *Trichoderma* and *Fusarium*, where the presence/absence of aegerolysin genes between species was detected.

Phylogenetic analyses of aegerolysins reveal considerable incongruence between gene and species trees (Butala et al. 2017), which, together with their uneven taxonomic distribution, suggests that horizontal gene transfer events contribute to the evolutionary history of aegerolysins (Moran et al. 2012). Our phylogenetic analysis is in line with this hypothesis, as we detected three cases with incongruencies between gene and species tree that are difficult to explain with lineage-specific gene losses. Similar patterns of taxonomic distribution and phylogenetic clustering are reported for two other pore-forming proteins, aerolysins and actinoporins (Szczesny et al. 2011; Moran et al. 2012). Although the amino acid sequence identities between the identified *Trichoderma* aegerolysins are low, as reported for other aegerolysins (Moran et al. 2012; Butala et al. 2017), they cluster together in a monophyletic group in the phylogenetic analysis.

Certain fungal aegerolysins are suggested to be involved in sexual development and fungal virulence (Novak et al. 2015; Butala et al. 2017). We therefore tested the involvement of *T. atroviride* AGL1 in these functions by investigating regulatory patterns and by generating *agl1* deletion strains. The high expression of *agl1* in conidiating mycelia indicates a role for AGL1 in conidial development. This is consistent with the expression pattern of aegerolysin genes in *A*. *niger* where expression of *nigA1* and *nigA2* is induced in conidiating mycelia (Novak et al. 2019). The role of AGL1 in conidiation is further verified by the reduced conidial production in the Δ*agl1* strains compared with the *T*. *atroviride* WT. The reduced conidial production in Δ*agl1* strains is in contrast to the previous findings involving *A*. *niger* and *A*. *fumigatus*, in which deletion of *nigA1* and *nigA2* and *aspHS* resulted in mutants with no difference in conidial production (Wartenberg et al 2011; Novak et al. 2019). The exact mechanistic connection between AGL1 and asexual development in *T. atroviride* requires further investigation. However, we may speculate that the reduced conidiation in Δ*agl1* strains is the result of disturbed interactions between the aegerolysin and a membrane lipid (Novak et al. 2019; Panevska et al. 2019). The role of membrane lipids and lipid-mediated cellular signaling in conidiation of filamentous fungi is well known (Li et al. 2007; Rhome and Del Poeta 2009; Hubar et al. 2019).

Expression of *agl1* is abolished during hyphal growth in iron-limiting medium, suggesting that iron is a positive regulator of *agl1* expression in *T*. *atroviride*. A similar result is reported from *A*. *fumigatus* where expression of the *aspHS* aegerolysin gene is significantly reduced during iron-limiting condition (McDonagh et al. 2008). On the other hand, the expression of *nigA1* and *nigA2* in *A*. *niger* is not affected under similar condition, indicating species-specific adaptation of aegerolysin gene regulation (Novak et al. 2019). Several putative iron-dependent dioxygenases are differentially regulated during conidial development in *T. reesei* (Metz et al. 2011), which may suggest a possible connection between *agl1* regulation by iron and during conidial development.

Expression of *agl1* was not detected in *T. atroviride* during interactions with neither *B. cinerea* nor *R. solani*, suggesting that AGL1 is dispensable for mycoparasitism. Accordingly, we were not able to detect any phenotypic defect regarding interspecific interactions in the Δ*agl1* strains. However, our results from the secretion assay suggest a role of AGL1 in antagonism, as higher growth rate of *B*. *cinerea* and *R*. *solani* was observed in growth media previously colonized by conidiated mycelia of Δ*agl1* strains, compared with the lower growth rate on media colonized by the WT. This indicates the absence of a secreted factor with growth-inhibiting property in the Δ*agl1* strains. This secreted factor can be AGL1 itself, or another factor whose expression is indirectly affected by the *agl1* deletion. The antagonistic effect was more severe towards the basidiomycete *R*. *solani* compared to the ascomycete *B*. *cinerea*. This is in agreement with the previous reports where external application of a purified haemolysin, lebbeckalysin, from *Albizia lebbeck* seeds reduced the mycelial growth of *R*. *solani*, while no effect in mycelial growth of *F*. *oxysporum*, *Helminthosporium maydis*, *Valsa mali,* or *Mycosphaerella arachidicola* was found (Lam and Ng 2011). Similar selective effects on insects are reported for aegerolysins from *P*. *ostreatus* where feeding of leaf discs treated with OlyA, PlyA2, and EryA demonstrated their interaction with pleurotolysin B (PlyB), resulting in selective toxicity towards certain insect pests including western corn rootworm (*Diabrotica virgifera virgifera*) larvae and adults and Colorado potato beetle larvae (*Leptinotarsa decemlineata*), but not towards mealworm (*Tenebrio molitor*), spotted wing Drosophila (*Drosophila suzukii*), grain aphid (*Sitobion avenae*), or greater wax moth (*Galleria mellonella*) (Panevska et al. 2019). This selective insecticidal activity was attributed to the physiological differences between the species tested (Panevska et al. 2019).

Somewhat surprisingly, expression of *agl1* was induced in *T. atroviride* during growth on cell wall material from *R. solani*, compared with growth on glucose- or chitin-containing media or during interaction with *R. solani*. As aegerolysins interact with membrane lipids (Novak et al. 2019; Panevska et al. 2019), it is possible that lipids present in the *R. solani* cell wall fraction (Kang et al. 2018) may act as an inducer of *agl1* expression. Certain pore-forming proteins are implicated in nutrient acquisition, for example hydralysin from the cnidarian *Chlorohydra viridissima* (Sher et al. 2008) and a pore-forming lytic protein from the hematophagous insect *Triatoma infestans* (Amino et al. 2002). However, the lack of difference in growth rate between WT and Δ*agl1* strains on *R. solani* cell wall medium shows that AGL1 is dispensable for this phenotype.

In conclusion, we identified and characterized the *agl1* aegerolysin gene in the mycoparasitic fungus *T*. *atroviride*. Our results indicate that AGL1 is involved in conidiation and antagonism.

## Electronic supplementary material

Below is the link to the electronic supplementary material.

Supplementary file1 (PDF 140 kb)

Supplementary file2 (PDF 1243 kb)

Supplementary file3 (XLSX 21 kb)

Supplementary file4 (PDF 107 kb)

Supplementary file5 (PDF 177 kb)
