# Supplementary material for: Functional characterization of the AGL1 aegerolysin in the mycoparasitic fungus Trichoderma atroviride reveals a role in conidiation and antagonism
Source: Mol Genet Genomics. 2020 Oct 14;296(1):131–40. doi: 10.1007/s00438-020-01732-3 (PMC7840653; doi:10.1007/s00438-020-01732-3)
Supplement: Supplementary file 1 — Supplementary file1 (PDF 140 kb) [file 438_2020_1732_MOESM1_ESM.pdf]

Supplementary figure S1

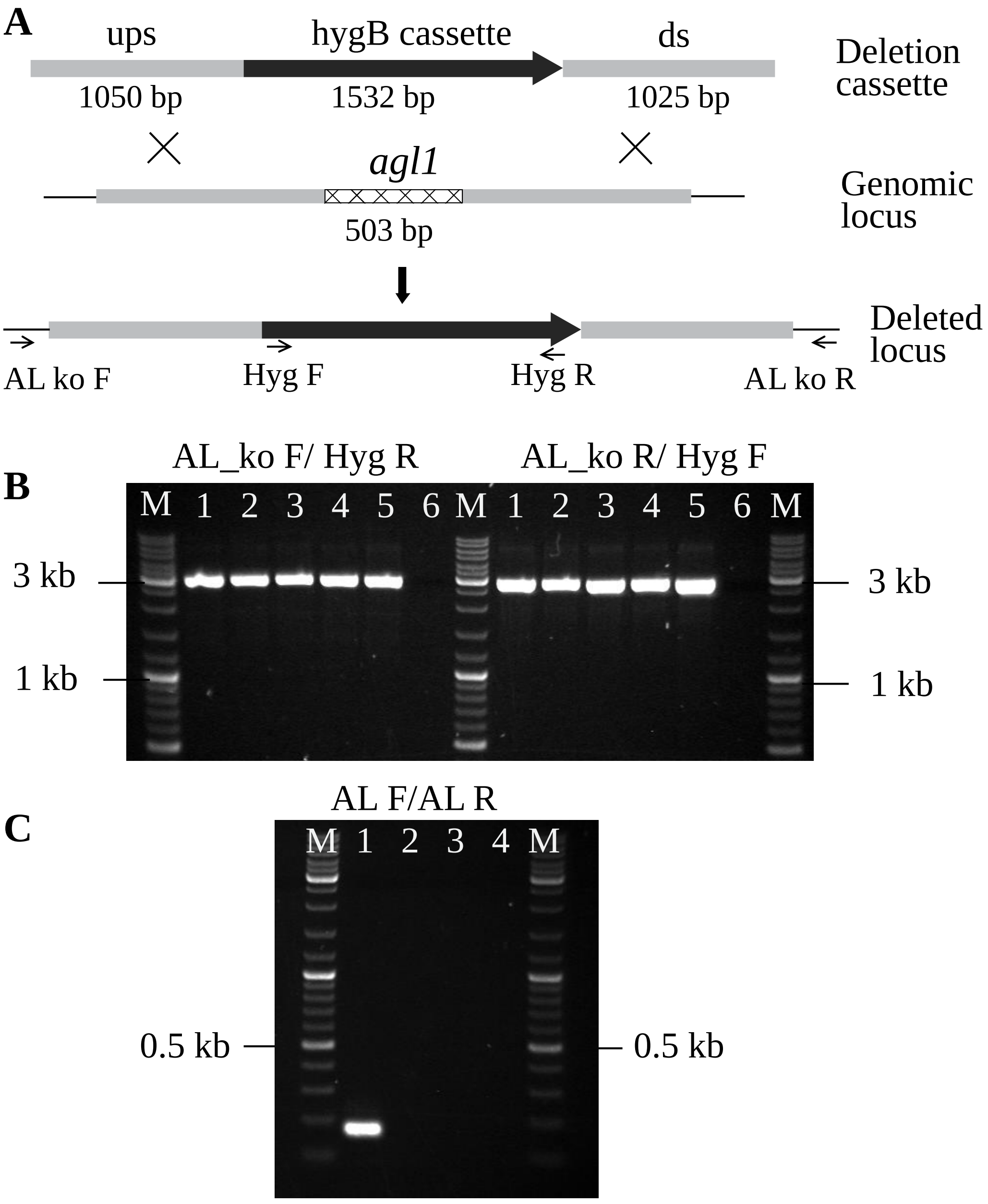

Figure S1. Construction of *agl1* deletion cassettes and characterization of mutant strains using PCR and RT-PCR. (A) Organisation of *agl1* locus in WT and mutant strains of *T. atroviride*. The *agl1* was replaced by hygB cassette by homologous recombination resulting in generation of Δ*agl1* strains. (B) PCR verification of Δ*agl1* strains using primers located in the hygB cassette in combination with primers located upstream and downstream from the deletion cassette. PCR products of ~2.9 were expected from a correct gene replacement. M, gene ruler DNA ladder mix; 1-5, independent Δ*agl1* strains; 6 WT strain. (C) RT-PCR analysis of *agl1* gene expression in WT and Δ*agl1* strains using *agl1* specific primers. M, gene ruler DNA ladder mix; 1, WT; 2-4, independent Δ*agl1* strains. Primer combinations used for PCR and RT-PCR are given above the images. The small arrow heads indicate the location of primers used to construct the deletion cassette and analysis of mutants using PCR.
