# Supplementary material for: Functional characterization of the AGL1 aegerolysin in the mycoparasitic fungus Trichoderma atroviride reveals a role in conidiation and antagonism
Source: Mol Genet Genomics. 2020 Oct 14;296(1):131–40. doi: 10.1007/s00438-020-01732-3 (PMC7840653; doi:10.1007/s00438-020-01732-3)
Supplement: Supplementary file 2 — Supplementary file2 (PDF 1243 kb) [file 438_2020_1732_MOESM2_ESM.pdf]

Supplementary figure S2

Botrytis cinerea

A

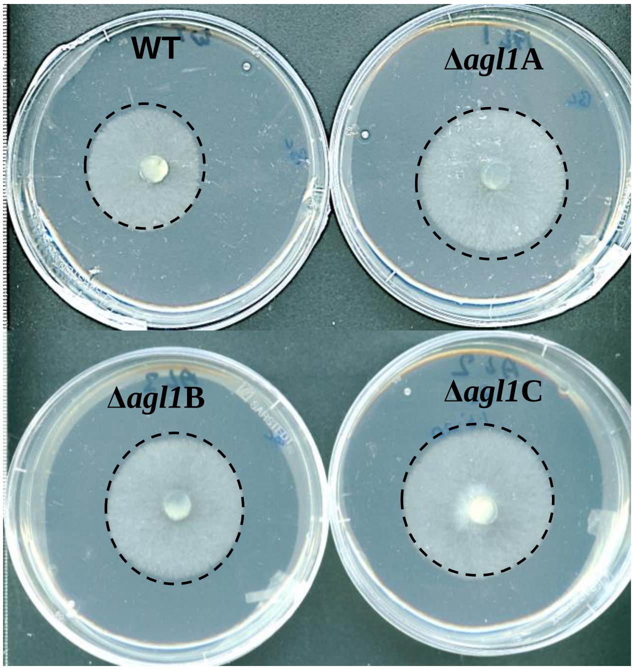

B

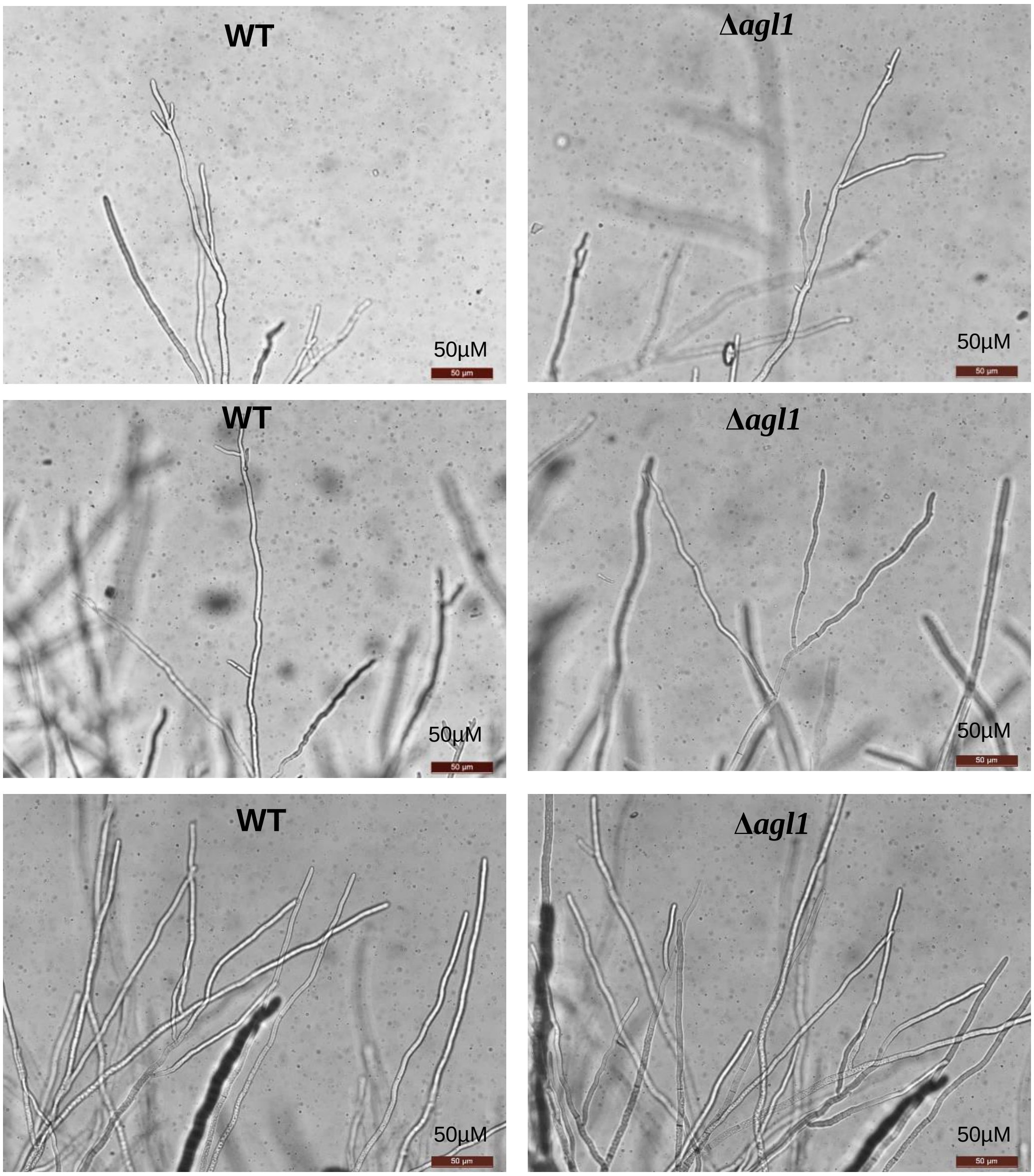

Rhizoctonia solani

C

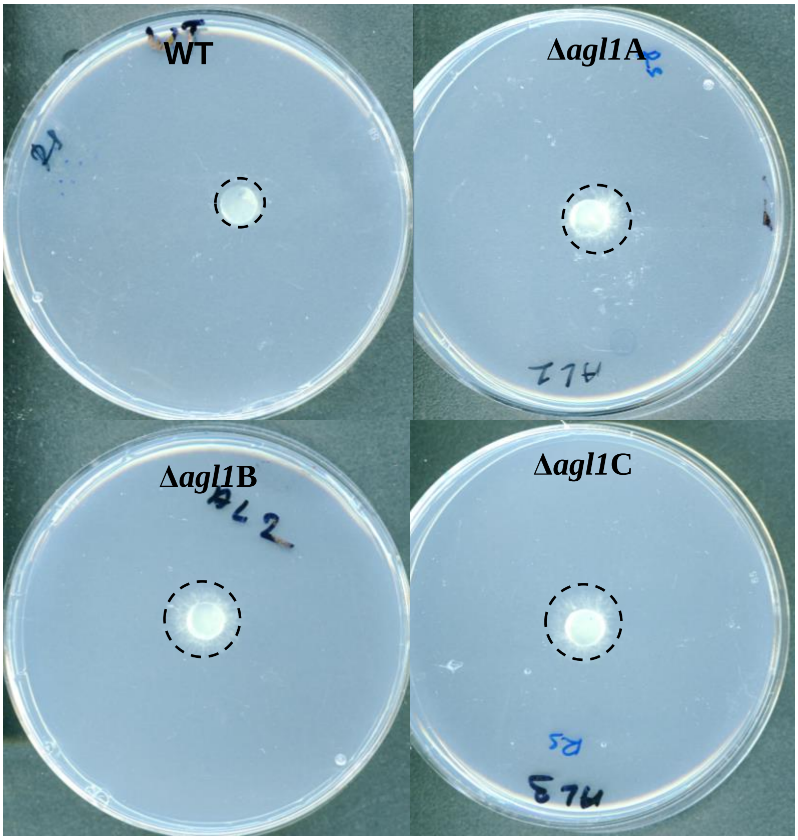

D

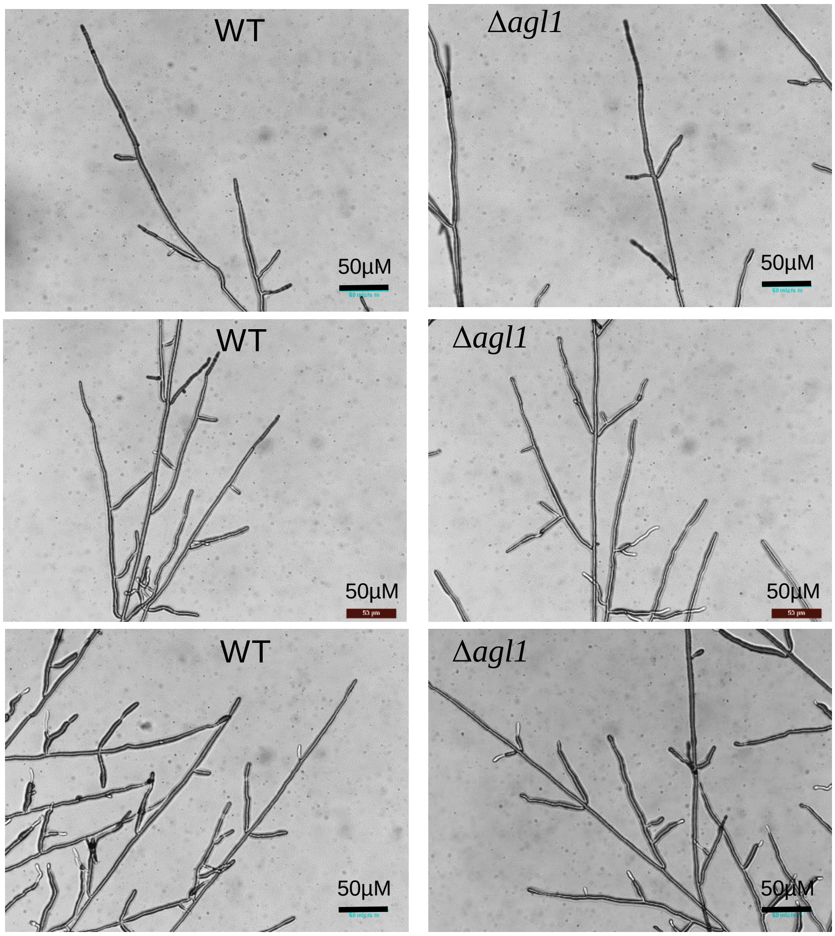

Supplementary figure S2: Secretion assay to analyse the antagonistic properties of *Trichoderma atroviride* WT and Δ*agl1* strains. Agar plugs were inoculated on PDA plates covered with cellophane membrane and incubated at 25°C. After 3 days of inoculation, the colony of *T. atroviride* strains was removed together with the cellophane disc. Plates were re-inoculated with a *Botrytis cinerea* or *Rhizoctonia solani* agar plug and incubated at 25°C. The experiment was performed in three biological replicates and photographs of representative plates were taken three days post inoculation.

A and C. Colony morphology of *B. cinerea* and *R. solani* grown on PDA plates pre-inoculated with *T. atroviride* WT or Δ*agl1* strains.

B and D. Microscopic analysis of *B*. *cinerea* and *R*. *solani* mycelia grown on PDA plates pre-inoculated with *T. atroviride* WT or Δ*agl1* strains. Mycelial morphology was analysed using a Zeiss Axioplan microscope (Thornwood, NY) equipped with Leica application suite version 3.6.0.
