# Supplementary material for: Functional characterization of the AGL1 aegerolysin in the mycoparasitic fungus Trichoderma atroviride reveals a role in conidiation and antagonism
Source: Mol Genet Genomics. 2020 Oct 14;296(1):131–40. doi: 10.1007/s00438-020-01732-3 (PMC7840653; doi:10.1007/s00438-020-01732-3)
Supplement: Supplementary file 4 — Supplementary file4 (PDF 107 kb) [file 438_2020_1732_MOESM4_ESM.pdf]

Table S2. Growth rate (mm / day) of *Trichoderma atroviride* WT and *agl1* deletion strains.

Medium	*T. atroviride* WT	Δ*agl1*A	Δ*agl1*B	Δ*agl1*C
PDA	21.3 ± 014[a]	21.1 ± 0.29[a]	21.3 ± 0.25[a]	21.0 ± 0.25[a]
RsCW	7.7 ± 14[a]	7.8 ± 29[a]	8.0 ± 25[a]	8.0 ± 43[a]

RsCW = SMS medium supplemented with *Rhizoctonia solani* cell wall.

Same letters indicate no statistically significant differences ($P \leq 0.05$) within the experiments based on Fisher's exact test.
Standard deviation based on three biological replicates.
