# Supplementary material for: Functional characterization of the AGL1 aegerolysin in the mycoparasitic fungus Trichoderma atroviride reveals a role in conidiation and antagonism
Source: Mol Genet Genomics. 2020 Oct 14;296(1):131–40. doi: 10.1007/s00438-020-01732-3 (PMC7840653; doi:10.1007/s00438-020-01732-3)
Supplement: Supplementary file 5 — Supplementary file5 (PDF 177 kb) [file 438_2020_1732_MOESM5_ESM.pdf]

Table S3. Growth (in cm) of *Botrytis cinerea* and *Rhizoctonia solani* during dual culture assay against *Trichoderma atroviride* WT and *agl1* deletion strains.

Medium	*T. atroviride* WT	Δ*agl1*A	Δ*agl1*B	Δ*agl1*C
Botrytis cinerea				
PDA	4.57 ± 0.06^a	4.63 ± 0.06^a	4.47 ± 0.15^a	4.60 ± 0.10^a
RsCw	3.20 ± 0.17^a	3.17 ± 0.15^a	3.13 ± 0.15^a	3.10 ± 0.17^a
Rhizoctonia solani				
PDA	4.13 ± 0.06^a	4.33 ± 0.21^a	4.30 ± 0.10^a	4.23 ± 0.15^a
RsCw	5.17 ± 0.15^a	5.47 ± 0.23^a	5.17 ± 0.06^a	5.23 ± 0.15^a

RsCW: SMS medium supplemented with *Rhizoctonia solani* cell wall.

Same letters indicate no statistically significant differences ($P \leq 0.05$) within the experiments based on Fisher's exact test.
Standard deviation based on four biological replicates.
